# Epicardial Adipose Tissue and Development of Atrial Fibrillation (AFIB) and Heart Failure With Preserved Ejection Fraction (HFpEF)

**DOI:** 10.7759/cureus.46153

**Published:** 2023-09-28

**Authors:** Sarmad Zain, Talha Shamshad, Ahmad Kabir, Ahmad Ali Khan

**Affiliations:** 1 Internal Medicine, Nishtar Medical University, Multan, PAK; 2 Pulmonology & Critical Care, Ch. Pervaiz Elahi Institute of Cardiology Multan, Multan, PAK; 3 Cardiology, Ch. Pervaiz Elahi Institute of Cardiology Multan, Multan, PAK

**Keywords:** pat, pericardial adipose tissue, eat thickness, la-eav, eat volume, hfpef, atrial fibrillation, epicardial adipose tissue

## Abstract

Epicardial adipose tissue (EAT) has been associated with the development of many cardiovascular abnormalities, of which the development of atrial fibrillation (AFIB) in this group of patients is not an uncommon finding. Several mechanisms have been proposed to explain the role of EAT in the development of AFIB. It involves cardiac remodeling owing to the underlying fatty infiltration and the subsequent inflammation and fibrosis. This leads to the formation of ectopic foci that can lead to AFIB. Some studies propose that structural and valvular heart disease and increased hemodynamic stress further augment the development of AFIB in patients with underlying EAT. The degree of development of AFIB is also related to EAT thickness and volume. Therefore, EAT quantification can be used as an imaging technique to predict cardiovascular outcomes in these patients. Obesity also plays an important role in the development of AFIB both as an independent factor and by leading to adipose tissue deposition on the epicardial tissue. Understanding the pathophysiology of EAT is important as it can lead to the development of therapies that can target obesity as a risk factor for preventing AFIB. Some promising therapies have already been investigated for decreasing the risk of AFIB in patients with EAT. Dietary changes and weight loss have been shown to reduce the deposition of fat on epicardial tissue. Antidiabetic drugs and statin therapy have also shown promising results. Bariatric surgery has been shown to decrease EAT volume on echocardiography in obese patients.

## Introduction and background

Epicardial adipose tissue (EAT) is a metabolically active tissue present over the heart. It is often associated with the development of atrial fibrillation (AFIB) and heart failure with preserved ejection fraction (HFpEF). EAT parameters like total EAT volume, left arterial (LA)-EAT volume, and EAT thickness can sometimes be used as predictors of cardiovascular outcomes in these patients. The scope of this review is to outline the understanding of the underlying mechanism of this epicardial fat tissue leading to cardiac remodeling that subsequently leads to AFIB and heart failure. Moreover, we highlight the association between obesity and the presence of excessive EAT and obesity as an independent cardiovascular risk factor in these patients.

The mainstay investigation for quantifying EAT parameters is echocardiography. Once the patient has been stratified using these parameters, certain therapies and interventions can be started to target the reduction of the total EAT volume and thickness. Some studies have shown statistically significant reductions in EAT volume and their association with the cardiovascular outcomes in these patients. The implications of these therapies have also been highlighted in this review. These therapies and interventions include obesity reduction by weight loss and bariatric surgery, antidiabetic agents (particularly GLP-1R agonists and SGLT2 inhibitors), and statin therapy.

## Review

The deposition of adipose tissue on the epicardium has been associated with a number of complications in the cardiovascular system. This occurs primarily by the cardiac remodeling that the cardiac tissue undergoes under the influence of EAT deposition. The underlying mechanism of this cardiac remodeling is not completely understood, but it involves inflammation, fibrosis, and neural dysregulation of the affected tissue [1]. Most of the EAT in the heart is in the atrioventricular and interventricular grooves. It is primarily divided into pericoronary and myocardial EAT. Pericoronary EAT, as the name suggests, is located around or on the adventitia of the coronary arteries. Myocardial EAT is present over the myocardial tissue.

In normal states from neonatal life to adulthood, EAT generally has cardioprotective effects. There are several ways by which EAT provides this cardioprotective effect. The primary mechanism by which this happens is the protection of the myocardial tissue by EAT. EAT acts as a buffer and protects the cardiac tissue against high levels of fatty acids that can potentially have detrimental effects on the cardiac tissue. A protein called adipocyte fatty acid binding protein (FABP4) facilitates the intracellular transport of fatty acids from the epicardial fat tissue into the myocardium [2].

The expression of some adipokines such as adiponectin and adrenomedullin through this EAT transcriptome has some anti-inflammatory and antiatherogenic properties as well [3]. EAT is also considered a direct source of heat to the myocardial tissue and is believed to be cardioprotective in states of stress like ischemia or hypoxia [4]. In these high-stress states, myocardial tissue demand is increased, and the presence of EAT facilitates meeting this increasing energy demand.

However, with aging, EAT starts developing pathological changes. This is attributed mainly to a decrease in the brown-fat activity in EAT because of chronic stress like ischemic conditions and coronary artery disease [5]. Several factors have been identified as crucial contributors leading to cardiac tissue remodeling because of the presence of EAT. Adipose tissue is metabolically active and results in the release of pro-inflammatory molecules and cytokines. These cytokines can trigger inflammation leading to the migration of macrophages and generating T cell response. This inflammatory cascade gradually damages the cardiac tissue over time. It leads to excessive accumulation of fibrous connective tissue on the cardiac tissue and development of the subsequent fibrosis.

Studies have also shown a strong correlation between the presence of excessive EAT and coronary artery disease [6]. Overlying excessive EAT over time can lead to atherosclerosis. The presence of EAT leads to the activation of inflammatory pathways that cause atherosclerosis in the adjacent coronary arteries. This is especially true for the EAT present in the pericoronary adventitia. This adipose tissue contributes to the development of atherosclerotic plaques in the adjacent coronary arteries.

The underlying tissue shows dense infiltrates rich in macrophages, CD8+ T cells, and mast cells [7]. High concentrations of CD4+ T cells are also found in patients with EAT, particularly in the obese population [8]. EAT also leads to endothelial dysfunction, which further contributes to increased cardiovascular risk. This endothelial dysfunction is primarily governed by cysteine-rich hormones such as resistin, which are released directly from the overlying adipose tissue [9].

Chronic inflammation induced by EAT can also activate fibroblasts, leading to the deposition of collagen and an eventual decrease in cardiac contractility over time. This reduces the diastolic heart function and further increases the risk of the development of cardiovascular disease in the patients. van Woerden et al. conducted a study in a population of 105 people with heart failure with midrange and preserved ejection fraction (HFpEF). The study population had a mean age 72±8 years, with a 50% female population, and a mean left ventricular ejection fraction of 53±8%. This study concluded that the presence of EAT is associated with poor prognosis in heart failure patients both with midrange EF and HFpEF [10]. This further elaborates on the influence of EAT in causing structural remodeling of the cardiac tissue because of the process of increased underlying fibroblastic activity. Studies also show that the thickness of epicardial fat is also directly related to adverse outcomes in patients with heart failure. Increased thickness of epicardial fat tissue is seen in both obese and nonobese populations with congestive heart failure [11].

Excessive deposition of EAT can be found incidentally in patients undergoing workup for other cardiovascular conditions. Imaging techniques including echocardiography, cardiac multidetector CT, and cardiac MRI remain the mainstay of investigation for quantifying EAT and EAT-related parameters like total EAT volume, LA-EAT, and EAT thickness. EAT was first described on echocardiography by Iacobellis et al. They measured EAT thickness on the right ventricle from both parasternal long- and short-axis views and described this layer of tissue as an echo-free space above the right ventricle [12].

The aortic ring is usually used as a reference point for the parasternal long axis, and the papillary muscles are used as a reference for the short-axis views. EAT can sometimes also appear as an echo-dense space with areas of inflammation on echocardiography [13]. Echocardiography offers valuable insights into epicardial adipose tissue (EAT) quantification. Measurement of EAT thickness, evaluation of its echogenicity, and assessment of its relationship with other cardiac structures provide useful information. This information can be used both as an indicator of cardiovascular outcome and as a tool for cardiac risk stratification in certain patient groups. For instance, EAT is associated with poor prognosis in heart failure and AFIB patients.

Easy accessibility and cost-effectiveness make echocardiography an excellent choice for the imaging modality for EAT quantification. However, it has a few limitations which include intra- and interoperator variability and inability to measure the EAT volume [14]. EAT volume is best measured using cardiac multidetector CT or cardiac MRI [15]. Another added advantage of CT and MRI is the quantification of EAT in areas not visually accessible by transthoracic echocardiography. This is particularly important in visualizing deeper areas such as pericoronary EAT, which can be used to predict the risk of CAD due to EAT in these patients [4,8].

An important metric during CT imaging is the fat attenuation index (FAI), which measures the metabolic activity in perivascular EAT and can be used as a marker of the underlying inflammation in perivascular EAT [16]. This can be used to predict the risk of coronary artery disease in patients with high perivascular EAT. A study showed that elevated perivascular FAI values (with a threshold of ≥-70.1 HU) can serve as a marker for heightened cardiac mortality risk [17].

The presence of EAT has also been associated with increased recurrence of AFIB after catheter ablation [18]. Two meta-analyses have been conducted on the subject that showed the association between the recurrence of AFIB after catheter ablation based on various parameters of quantifying EAT. These parameters are total EAT volume, LA-EAT volume, and EAT thickness. The first meta-analysis, which used data collected till 2018, showed that LA-EAT, EAT thickness, and total EAT volumes were increased in patients with recurrent episodes of AFIB after ablation [19]. This study, however, did not adjust for other risk factors like the relative risk (RR) and hazard ratio (HR) of relevant epicardial fat tissue parameters. Another meta-analysis, conducted in 2022, brought into account these factors and showed a higher association between the presence of EAT and the recurrence of AFIB in patients after catheter ablation. This study also showed that the association was higher in the Asian population, younger patients with AFIB, and long-term follow-up (>1 year) [20].

A body mass index of more than 30 is considered obese, and it is characterized by the buildup of extra adipose tissue throughout the body, including around organs, within muscles, within the bone marrow, and beneath the skin [21]. Obesity is now the second-highest population-attributable risk for AF, trailing only hypertension as the main cause of AFIB in the general population, although its significance is expanding [22]. Obese people are more likely to experience HFpEF and AFIB. The development of both AFIB and HFpEF in obese patients may be influenced by the increased thickness of epicardial fat and inflammation in this fat layer [23].

In obesity, fat tissue contains activated macrophages, causing inflammation. Weight loss reduces these macrophages and inflammation [24,25]. Lean mice have anti-inflammatory CD4+ regulatory T cells [26], while obese mice have pro-inflammatory CD8+ T cells that activate harmful macrophages [27]. Lean mice show M2 anti-inflammatory macrophages, while obese mice have M1 pro-inflammatory macrophages [28]. According to a meta-analysis comprising multiple observational studies, the degree of connection between AFIB and epicardial fat was much stronger than that between AFIB and either abdominal or total adiposity, emphasizing the potential mechanistic and clinical significance of epicardial fat in AFIB patients [29].

Targeting the immunochemical pathways of epicardial adipose tissue-induced AFIB is a topic of ongoing research. The use of statins in relation to reducing the epicardial adipose tissue helps in abolishing AFIB. Epicardial fat resists weight loss programs fairly well [30]. Therefore, the small amount of weight reduction that is normally achieved by restricting calories has little impact on the adipose tissue that lines the heart [31] and less impact on AFIB [32]. Conversely, significant weight reduction (e.g., through bariatric surgery) can reduce the size and inflammation of epicardial fat [33,34]. Significant weight reduction has also been shown in both observational studies and randomized controlled trials to ease the symptoms of AFIB or generate sinus rhythm in those with AFIB that has already developed [35,36].

Some trials have demonstrated a reduction in the incidence of significant cardiovascular events with GLP-1R agonist and SGLT2 inhibitor treatments, with a magnitude of effects indicating mechanisms beyond gains in glycemic control; however, the exact nature of these processes is not yet completely understood [37-42].

GLP-1R agonists are used to treat obesity and type 2 diabetes mellitus and have additional cardiovascular advantages beyond glycemic benefits [38-40]. One of the GLP-1R agonist liraglutide's non-glycemic effects has been proposed as a reduction in visceral fat [41]. The GLP-1R agonists lower epicardial adipose tissue thickness more significantly than overall weight loss in patients with type 2 diabetes and obesity [42-45]. Inducing brown fat transformation, decreasing local fat tissue development, improving fat consumption, and modulating the renin-angiotensin axis are all possible effects of activating the GLP-1R [46-48]. Epicardial adipose tissue has been found to express GLP-1R, and this finding supports a possible direct effect of GLP-1R agonists on it [49].

The pathological mRNA profiles of omental and epicardial fat are largely similar; this may be partially because of the infiltration of macrophages within the epicardial fat. Patients on statins exhibited noticeably reduced IL-6 mRNA epicardial expression when compared to control abdominal depots [50]. Another study demonstrated that lower levels of EAT-secreted inflammatory mediators and reduced EAT thickness were both substantially correlated with statin therapy. Interestingly, EAT thickness and its pro-inflammatory state showed a strong association [51]. Table 1 shows a summary of the meta-analyses and systematic reviews conducted on the role of certain interventions and therapies on EAT-relevant outcomes.

**Table 1:** A Summary of Studies Conducted on EAT-Targeted Interventions/Therapy and Relevant Outcomes EAT: Epicardial Adipose Tissue; PAT: Pericardial Adipose Tissue; ES: Effect Size; CI: Confidence Interval

Author(s)	Year	Findings	Summary
Takao et al. [52]	2020	Initially, EAT volumes in the dapagliflozin and conventional treatment groups were similar, measuring 113±20 and 110±27 cm^3^, respectively. After six months, the dapagliflozin group exhibited a significant decrease in EAT volume from baseline. Moreover, the reduction in EAT volume within the dapagliflozin group surpassed that of the conventional treatment group (−15.2±12.8 vs. 3.0±11.9 cm^3^, p = 0.01).	Dapaglifozin significantly reduced EAT volume in comparison to the conventional treatment group at a six-month interval.
Soucek et al. [53]	2016	Among the 79 participants enrolled, 38 were administered atorvastatin and 41 received a placebo. No noteworthy distinctions in baseline traits were observed between the study cohorts. Individuals assigned atorvastatin displayed a noteworthy decrease in median EAT volume following a three-month treatment [change −4.6 (−8.9 to 1.3) cm^3^, p<0.05].	Compared to the placebo group, patients in the Atorvastatin group had a statistically significant reduction in EAT volume at a three-month interval.
Launboet al. [54]	2020	The meta-analysis focused on changes in EAT volume, as few controlled studies reported PAT changes (n=3) or total cardiac adipose tissue volume changes (n=1). Notably, weight-loss interventions showed a significant reduction in EAT volume compared to control interventions (pooled effect size, ES=−0.89, 95% CI:−1.23 to −0.55, P<0.001). When comparing exercise training to control, exercise had a significant positive effect (ES:−1.11, 95% CI:−1.57 to −0.65, P<0.001), and pharmaceutical interventions also significantly reduced EAT volume (ES:−0.79, 95% CI:−1.37 to −0.21, P<0.0072).	Weight-loss interventions demonstrated a noteworthy decrease in EAT volume in comparison to control interventions. Similarly, pharmaceutical interventions led to a significant reduction in EAT volume.

These studies show that interventions like weight loss and therapies, particularly antidiabetic agents and statins, have a statistically significant impact on lower EAT volume and thickness. Over the course of time, statins were found to reduce epicardial adipose tissue regardless of how much LDL cholesterol was lowered. The observed decrease in metabolic activity in epicardial adipose tissue due to statins may have been caused by a decrease in cellularity, circulation, or inflammatory processes, as indicated by the beneficial impact on epicardial adipose tissue. Further understanding of the mechanisms of EAT leading to cardiac remodeling can be pivotal in forming new targeted therapies. These therapies can not only reduce the chance of conditions like AFIB and HFpEF in these patients due to the underlying EAT but can also provide overall cardiovascular benefits in these patients.

## Conclusions

Understanding the pathophysiological link between EAT and the emergence of AFIB and HFpEF holds therapeutic potential. Alongside interventions like dietary modifications and weight loss, treatments involving antidiabetic agents and statin therapy have shown noteworthy results in certain clinical trials. Notably, these therapies have demonstrated statistically significant reductions in overall EAT volume, LA-EAT volume, and EAT thickness among these patients. This decrease in EAT is not only an indicator of better cardiovascular outcomes but also correlates with reduced recurrence of conditions such as AFIB in this patient group.
